# Bis(2,2′-bipyridyl-κ^2^ *N*,*N*′)chloridonickel(II) nitrate trihydrate

**DOI:** 10.1107/S1600536814009064

**Published:** 2014-04-26

**Authors:** Mehdi Boutebdja, Adel Beghidja, Chahrazed Beghidja, Zouaoui Setifi, Hocine Merazig

**Affiliations:** a Unité de Recherche de Chimie de l’Environnement et Moléculaire Structurale, (CHEMS), Faculté des Sciences Exactes, Département de Chimie, Université de Constantine 1, 25000 Constantine, Algeria; b Département de Technologie, Faculté de Technologie, Université 20 Août 1955-Skikda, BP 26, Route d’El-Hadaiek, Skikda 21000, Algeria

## Abstract

In the title hydrated salt, [NiCl(C_10_H_8_N_2_)_2_](NO_3_)·3H_2_O, the Ni^2+^ ion is coordinated by two 2,2′-bipyridyl (2,2′-bpy) ligands and a chloride ion in a trigonal–bipyramidal geometry. The chloride ion occupies an equatorial site and the dihedral angle between the 2,2′-bpy ring systems is 72.02 (6)°. In the crystal, the components are linked by C—H⋯O and O—H⋯O hydrogen bonds and aromatic π–π stacking inter­actions [shortest centroid–centroid separation = 3.635 (2) Å], generating a three-dimensional network.

## Related literature   

For the isotypic copper complex, see: Harrison *et al.* (1981 ▶); Liu *et al.* (2004 ▶). For related structures, see: Martens *et al.* (1996 ▶); Gao & Li (2009 ▶)
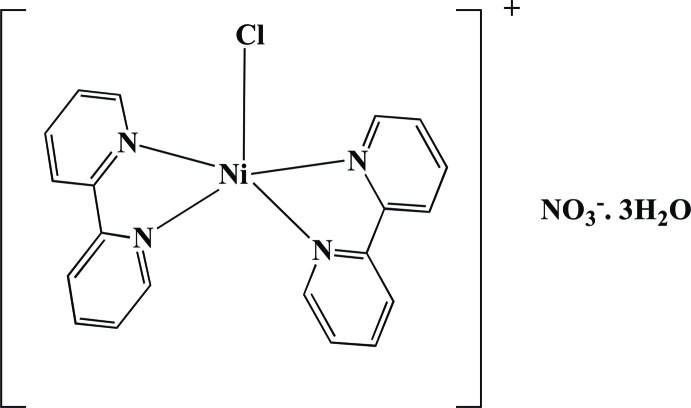


## Experimental   

### 

#### Crystal data  

[NiCl(C_10_H_8_N_2_)_2_](NO_3_)·3H_2_O
*M* *_r_* = 522.57
Monoclinic, 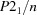

*a* = 8.2341 (2) Å
*b* = 21.1920 (5) Å
*c* = 13.1284 (4) Å
β = 99.722 (1)°
*V* = 2257.97 (10) Å^3^
*Z* = 4
Mo *K*α radiation
μ = 1.03 mm^−1^
*T* = 296 K
0.15 × 0.13 × 0.10 mm

#### Data collection  

Bruker APEXII CCD diffractometer
21125 measured reflections
5177 independent reflections
3811 reflections with *I* > 2σ(*I*)
*R*_int_ = 0.034

#### Refinement  

*R*[*F* ^2^ > 2σ(*F* ^2^)] = 0.044
*wR*(*F* ^2^) = 0.127
*S* = 1.01
5177 reflections
298 parameters
9 restraints
H-atom parameters constrained
Δρ_max_ = 0.47 e Å^−3^
Δρ_min_ = −0.47 e Å^−3^

### 

Data collection: *APEX2* (Bruker, 2006 ▶); cell refinement: *SAINT* (Bruker, 2006 ▶); data reduction: *SAINT*; program(s) used to solve structure: *SHELXS97* (Sheldrick, 2008 ▶); program(s) used to refine structure: *SHELXL97* (Sheldrick, 2008 ▶); molecular graphics: *ATOMS* (Dowty, 1995 ▶); software used to prepare material for publication: *WinGX* publication routines (Farrugia, 2012 ▶).

## Supplementary Material

Crystal structure: contains datablock(s) global, I. DOI: 10.1107/S1600536814009064/hb7220sup1.cif

Structure factors: contains datablock(s) I. DOI: 10.1107/S1600536814009064/hb7220Isup2.hkl

CCDC reference: 998760

Additional supporting information:  crystallographic information; 3D view; checkCIF report

## Figures and Tables

**Table 1:** Selected bond lengths (Å)

Ni1—Cl1	2.3035 (9)
Ni1—N1	1.989 (2)
Ni1—N2	2.088 (2)
Ni1—N3	2.107 (2)
Ni1—N4	1.983 (2)

**Table 2:** Hydrogen-bond geometry (Å, °)

*D*—H⋯*A*	*D*—H	H⋯*A*	*D*⋯*A*	*D*—H⋯*A*
O1*W*—H1*W*⋯O3*W* ^i^	0.81	2.29	2.876 (6)	129
O1*W*—H2*W*⋯O2^ii^	0.83	2.18	2.934 (7)	151
O2*W*—H3*W*⋯O2^ii^	0.84	1.90	2.723 (7)	166
O2*W*—H4*W*⋯Cl1^i^	0.83	2.47	3.245 (4)	155
O3*W*—H5*W*⋯O2*W* ^iii^	0.85	1.88	2.699 (6)	161
O3*W*—H6*W*⋯O1^iv^	0.83	2.03	2.839 (7)	165
C14—H14⋯O2*W*	0.93	2.56	3.424 (5)	155
C18—H18⋯O1*W*	0.93	2.39	3.257 (6)	156

Symmetry codes: (i) 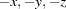; (ii) 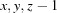; (iii) 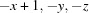; (iv) 
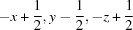
.

**Fig. 1.**
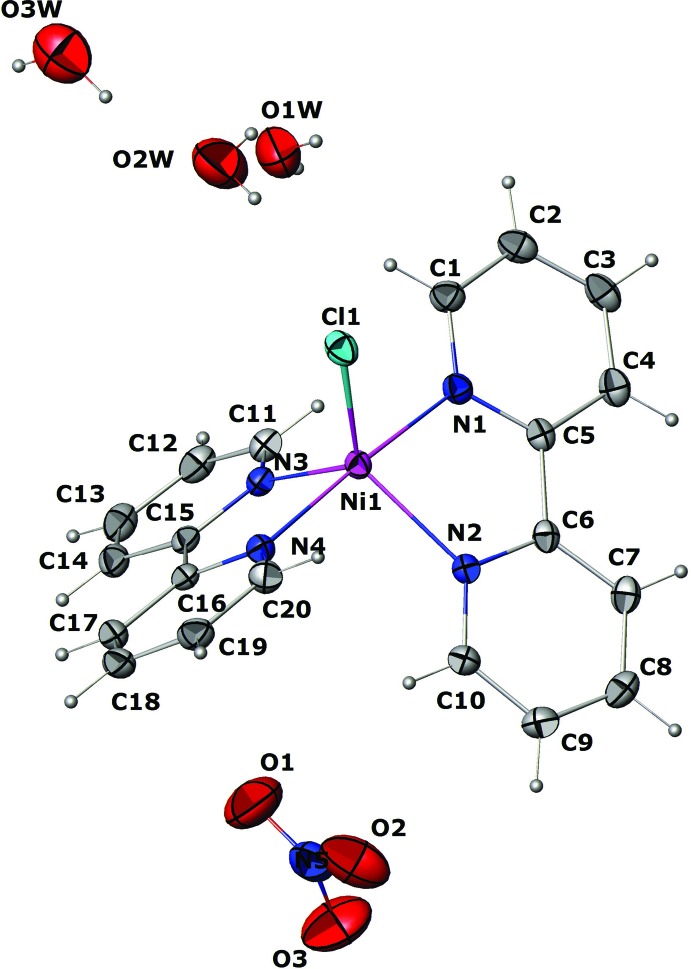
ORTEP view of the title compound with displacement ellipsoids for non-H atoms drawn at the 30% probability level.

**Fig. 2.**
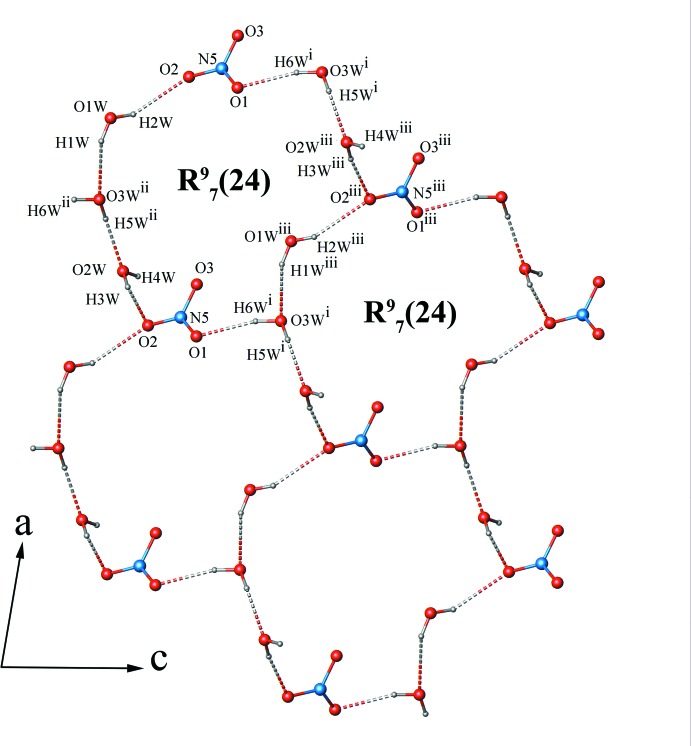
The two-dimensional water-nitrate framework parallel to ac plane, and the aggregation of R9 7(24)[Symmetry codes: (i) -x, -y, -z; (ii) x, y, z - 1; (iii) -x + 1, -y, -z]

**Fig. 3.**
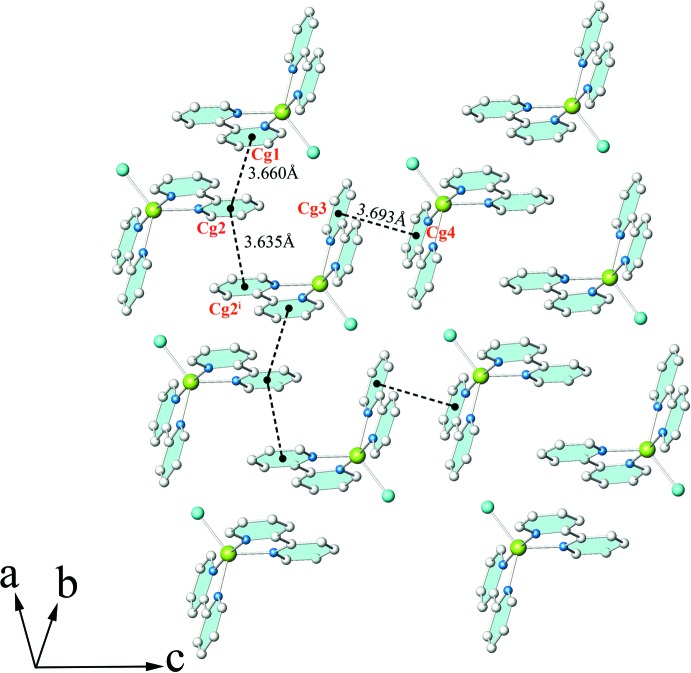
Part of the crystal structures, showing the [pi]-[pi] stacking interaction [Symmetry codes: (i) 1 - x, -y, -z]

**Fig. 4.**
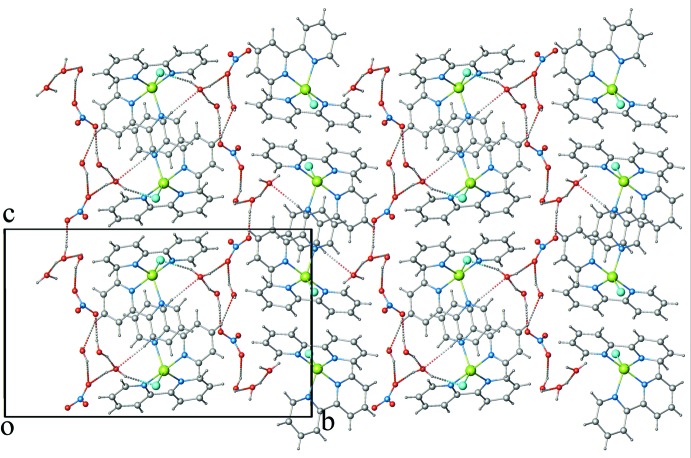
Packing diagram of the supramolecular edifice showing hydrogen bonds as dashed lines

## supplementary crystallographic information

### 1. Comment

The molecular structure of the title complex is shown in (Fig.1), The title compound is isostructural with the copper analogue (Harrison *et al.*, 1981; Liu *et al.*, 2004), crystalize in the monoclinic space group P2_1_/n. The Ni(II) atom is five-coordinate and displays a distorted trigonal-bipyramidal coordination geometry with four N atoms from the two chelating 2,2'-bipyridine molecules and one chloride ion. The basal plane defined by the atoms (N1 N3 Cl1). The apical positions are occupied by the N2 and N4 atoms [N2—Ni1—N4 = 175.09 (10)°]. The Ni—N bond lenghts (table 1) are in normal range [Ni1—N1 = 2.086 (3), Ni1—N2 = 1.984 (3), Ni1—N3 = 2.108 (3), Ni1—N4 = 1.983 (3), Ni1—Cl1 = 2.3032 (10)]. In the crystal structure, the components are linked by weak C—H···O and medium O—H···O hydrogen bonds. Water molecules are further hydrogen-bond-interacting with the nitrate anion to complete a two-dimensional water-nitrate framework parallel to (101)which can be described by the graph set R97(24) (Fig. 2). Thus, the discrete [Ni(bpy)_2_Cl]^+^ was linked to each other through pi-pi stacking to form two-dimensional supramolecular coordinated polymer parallel to the *ac* plane with centroid–centroiddistances of *Cg*(1)—*Cg*(2) = 3.660 (2) Å, *Cg*(2)—*Cg*(2i) = 3.635 (2) Å and *Cg*(3)—*Cg*(4) = 3.693 (2) Å. (*Cg*(1) is the centroid of N4—C20 2,2'-bpy ring, *Cg*(2) is the centroid of N3—C15 2,2'-bpy ring, *Cg*(3) is the centroid of N2—C10 2,2'-bpy ring, *Cg*(4) is the centroid of N1—C5 2,2'-bpy ring) (Fig.3). These layers are connected to each other *via* a weak O—H···Cl and C—H···O hydrogen bond to form a three-dimensional network(Fig.4).

### 2. Experimental

Compound (1) was obtained from the reaction of MSA 'mercaptosuccinic acid' (0.15 g, 1 mmol) in pyridine and an ethanolic solution of Ni(NO3)2.6H2O (0.290 g, 1 mmole) After several minutes of stirring an ethanol solution containing 2,2'-Bipyridine hydrochloride (0.114 g, 0.5 mmol) was add. The solution was kept for several weeks at room temperature. Green crystals suitable for X-ray analysis were obtained (yield: 0.1 g, 10% on the basis of Ni(NO3)2.6H2O).

### 3. Refinement

Water hydrogen atoms were tentatively found in the difference density Fourier map and were refined with an isotropic displacement parameter 1.5 that of the adjacent oxygen atom. The O—H distances were restrained to be 0.9 Å within a standard deviation of 0.01 with *U*_iso_(H) = 1.5 *U*_eq_(O) and the H···H contacts were restraint to 1.40 Å with a standard deviation of 0.02. A l l other Hydrogen atoms were placed in calculated positions with C —H distances of 0.93–0.96 Å for aromatic H atoms with *U*_iso_(H) =1.2 *U*_eq_(C). Maximum and minimum residual electron densities were 0.47 e Å-3 (0.79 Å from Ni1) and -0.47 e Å-3 (0.70 Å from H3w), respectively.

### Crystal data

**Table:**

[NiCl(C_10_H_8_N_2_)_2_](NO_3_)·3H_2_O	*Z* = 4
*M**_r_* = 522.57	*F*(000) = 1080
Monoclinic, *P*2_1_/*n*	*D*_x_ = 1.537 Mg m^−^^3^
Hall symbol: -P 2yn	Mo *K*α radiation, λ = 0.71073 Å
*a* = 8.2341 (2) Å	µ = 1.03 mm^−^^1^
*b* = 21.1920 (5) Å	*T* = 296 K
*c* = 13.1284 (4) Å	Block, green
β = 99.722 (1)°	0.15 × 0.13 × 0.10 mm
*V* = 2257.97 (10) Å^3^	

### Data collection

**Table:**

Bruker APEXII CCD diffractometer	3811 reflections with *I* > 2σ(*I*)
Radiation source: Rotating Anode	*R*_int_ = 0.034
Graphite monochromator	θ_max_ = 27.5°, θ_min_ = 2.7°
Detector resolution: 18.4 pixels mm^-1^	*h* = −10→10
φ and ω scans	*k* = −25→27
21125 measured reflections	*l* = −16→17
5177 independent reflections	

### Refinement

**Table:**

Refinement on *F*^2^	9 restraints
Least-squares matrix: full	Hydrogen site location: mixed
*R*[*F*^2^ > 2σ(*F*^2^)] = 0.044	H-atom parameters constrained
*wR*(*F*^2^) = 0.127	W = 1/[Σ^2^(*F*O^2^) + (0.0647*P*)^2^ + 1.1593*P*] WHERE *P* = (*F*O^2^ + 2*F*C^2^)/3
*S* = 1.01	(Δ/σ)_max_ < 0.001
5177 reflections	Δρ_max_ = 0.47 e Å^−^^3^
298 parameters	Δρ_min_ = −0.47 e Å^−^^3^

### Special details

**Table:**

Geometry. Bond distances, angles *etc*. have been calculated using the rounded fractional coordinates. All su's are estimated from the variances of the (full) variance-covariance matrix. The cell e.s.d.'s are taken into account in the estimation of distances, angles and torsion angles
Refinement. Refinement on *F*^2^ for ALL reflections except those flagged by the user for potential systematic errors. Weighted *R*-factors *wR* and all goodnesses of fit *S* are based on *F*^2^, conventional *R*-factors *R* are based on *F*, with *F* set to zero for negative *F*^2^. The observed criterion of *F*^2^ > σ(*F*^2^) is used only for calculating -*R*-factor-obs *etc*. and is not relevant to the choice of reflections for refinement. *R*-factors based on *F*^2^ are statistically about twice as large as those based on *F*, and *R*-factors based on ALL data will be even larger.

### Fractional atomic coordinates and isotropic or equivalent isotropic displacement parameters (Å^2^)

**Table:**

	*x*	*y*	*z*	*U*_iso_*/*U*_eq_	
Ni1	0.26205 (4)	0.01955 (2)	0.25456 (2)	0.0416 (1)	
Cl1	0.04397 (10)	−0.00729 (4)	0.33507 (6)	0.0607 (3)	
N1	0.4089 (3)	−0.04491 (11)	0.33240 (17)	0.0468 (7)	
N2	0.4655 (3)	0.07571 (11)	0.31172 (16)	0.0446 (7)	
N3	0.2831 (3)	−0.01045 (10)	0.10432 (18)	0.0448 (7)	
N4	0.1319 (3)	0.08621 (11)	0.17169 (18)	0.0470 (7)	
C1	0.3683 (4)	−0.10552 (14)	0.3398 (3)	0.0574 (10)	
C2	0.4723 (4)	−0.14864 (15)	0.3945 (3)	0.0626 (11)	
C3	0.6237 (4)	−0.12862 (16)	0.4447 (3)	0.0660 (11)	
C4	0.6663 (4)	−0.06611 (15)	0.4387 (2)	0.0573 (10)	
C5	0.5564 (3)	−0.02455 (13)	0.3819 (2)	0.0430 (8)	
C6	0.5881 (3)	0.04365 (13)	0.37109 (19)	0.0422 (8)	
C7	0.7311 (4)	0.07368 (16)	0.4171 (2)	0.0547 (10)	
C8	0.7489 (4)	0.13756 (17)	0.4001 (3)	0.0663 (11)	
C9	0.6260 (4)	0.16938 (16)	0.3385 (3)	0.0664 (11)	
C10	0.4856 (4)	0.13736 (14)	0.2963 (2)	0.0559 (10)	
C11	0.3647 (4)	−0.06032 (14)	0.0758 (2)	0.0531 (10)	
C12	0.3716 (4)	−0.07369 (17)	−0.0257 (3)	0.0623 (11)	
C13	0.2912 (4)	−0.03406 (19)	−0.1010 (3)	0.0668 (13)	
C14	0.2078 (4)	0.01749 (16)	−0.0732 (2)	0.0591 (10)	
C15	0.2067 (3)	0.02868 (13)	0.0305 (2)	0.0455 (8)	
C16	0.1201 (3)	0.08294 (13)	0.0683 (2)	0.0452 (8)	
C17	0.0298 (4)	0.12715 (15)	0.0049 (2)	0.0578 (10)	
C18	−0.0500 (4)	0.17463 (15)	0.0485 (3)	0.0649 (11)	
C19	−0.0382 (4)	0.17781 (15)	0.1538 (3)	0.0641 (11)	
C20	0.0545 (4)	0.13259 (14)	0.2133 (3)	0.0570 (10)	
O1	−0.1473 (7)	0.2935 (3)	0.5504 (4)	0.181 (3)	
O2	−0.0983 (7)	0.2268 (2)	0.6708 (5)	0.165 (3)	
O3	0.0639 (6)	0.2366 (3)	0.5677 (5)	0.186 (3)	
N5	−0.0656 (6)	0.2527 (2)	0.5946 (4)	0.1009 (19)	
O1W	−0.2649 (5)	0.2447 (3)	−0.1506 (3)	0.167 (2)	
O2W	0.1269 (6)	0.1387 (2)	−0.2468 (3)	0.157 (2)	
O3W	0.5892 (5)	−0.1931 (2)	0.1556 (4)	0.162 (2)	
H1	0.26540	−0.11900	0.30650	0.0690*	
H2	0.44110	−0.19070	0.39770	0.0750*	
H3	0.69660	−0.15700	0.48230	0.0790*	
H4	0.76800	−0.05180	0.47240	0.0690*	
H7	0.81390	0.05130	0.45880	0.0660*	
H8	0.84400	0.15860	0.43040	0.0790*	
H9	0.63700	0.21210	0.32520	0.0800*	
H10	0.40120	0.15950	0.25540	0.0670*	
H11	0.41890	−0.08690	0.12680	0.0640*	
H12	0.42910	−0.10870	−0.04320	0.0750*	
H13	0.29350	−0.04220	−0.17030	0.0800*	
H14	0.15300	0.04450	−0.12340	0.0710*	
H17	0.02300	0.12480	−0.06640	0.0690*	
H18	−0.11170	0.20440	0.00660	0.0780*	
H19	−0.09130	0.20960	0.18430	0.0770*	
H20	0.06340	0.13450	0.28480	0.0680*	
H1W	−0.35430	0.25310	−0.13630	0.2350*	
H2W	−0.25270	0.23560	−0.21040	0.2350*	
H3W	0.06220	0.16910	−0.26290	0.2350*	
H4W	0.10210	0.10720	−0.28370	0.2350*	
H5W	0.66590	−0.16810	0.18080	0.2350*	
H6W	0.58870	−0.19980	0.09320	0.2350*	

### Atomic displacement parameters (Å^2^)

**Table:**

	*U*^11^	*U*^22^	*U*^33^	*U*^12^	*U*^13^	*U*^23^
Ni1	0.0427 (2)	0.0381 (2)	0.0418 (2)	0.0038 (1)	0.0004 (1)	0.0021 (1)
Cl1	0.0508 (4)	0.0743 (5)	0.0580 (4)	0.0074 (3)	0.0119 (3)	0.0144 (4)
N1	0.0505 (13)	0.0427 (12)	0.0468 (12)	0.0062 (10)	0.0067 (10)	0.0002 (10)
N2	0.0460 (12)	0.0459 (12)	0.0410 (11)	−0.0002 (10)	0.0044 (9)	0.0014 (9)
N3	0.0418 (12)	0.0455 (13)	0.0458 (12)	−0.0053 (9)	0.0034 (9)	−0.0034 (10)
N4	0.0462 (13)	0.0441 (12)	0.0485 (13)	0.0001 (10)	0.0019 (10)	0.0024 (10)
C1	0.0612 (19)	0.0449 (16)	0.0656 (19)	0.0035 (13)	0.0096 (15)	0.0001 (14)
C2	0.076 (2)	0.0447 (16)	0.070 (2)	0.0145 (15)	0.0208 (17)	0.0037 (15)
C3	0.077 (2)	0.059 (2)	0.0617 (19)	0.0286 (17)	0.0113 (17)	0.0113 (15)
C4	0.0539 (17)	0.0634 (19)	0.0536 (17)	0.0163 (14)	0.0061 (13)	−0.0010 (14)
C5	0.0443 (14)	0.0495 (15)	0.0363 (12)	0.0094 (11)	0.0099 (11)	−0.0016 (11)
C6	0.0406 (13)	0.0539 (15)	0.0327 (12)	0.0038 (11)	0.0083 (10)	−0.0033 (11)
C7	0.0436 (15)	0.068 (2)	0.0514 (16)	0.0049 (14)	0.0052 (12)	−0.0070 (14)
C8	0.0527 (18)	0.073 (2)	0.072 (2)	−0.0155 (16)	0.0070 (16)	−0.0124 (17)
C9	0.072 (2)	0.0550 (19)	0.072 (2)	−0.0128 (16)	0.0115 (17)	0.0027 (16)
C10	0.0607 (18)	0.0476 (16)	0.0570 (17)	−0.0035 (13)	0.0030 (14)	0.0079 (13)
C11	0.0480 (16)	0.0535 (17)	0.0575 (17)	−0.0036 (13)	0.0078 (13)	−0.0086 (14)
C12	0.0540 (18)	0.069 (2)	0.067 (2)	−0.0095 (15)	0.0193 (15)	−0.0218 (17)
C13	0.063 (2)	0.092 (3)	0.0486 (17)	−0.0199 (19)	0.0191 (15)	−0.0150 (17)
C14	0.0549 (18)	0.077 (2)	0.0447 (16)	−0.0145 (15)	0.0068 (13)	0.0020 (15)
C15	0.0365 (13)	0.0523 (16)	0.0466 (14)	−0.0130 (11)	0.0042 (11)	0.0011 (12)
C16	0.0377 (13)	0.0459 (14)	0.0499 (15)	−0.0113 (11)	0.0011 (11)	0.0054 (12)
C17	0.0538 (17)	0.0575 (18)	0.0575 (18)	−0.0092 (15)	−0.0042 (14)	0.0145 (15)
C18	0.0596 (19)	0.0498 (18)	0.079 (2)	−0.0016 (15)	−0.0061 (16)	0.0185 (16)
C19	0.0599 (19)	0.0436 (17)	0.086 (2)	0.0032 (14)	0.0045 (17)	0.0029 (16)
C20	0.0610 (18)	0.0479 (16)	0.0606 (18)	0.0047 (14)	0.0061 (14)	−0.0020 (14)
O1	0.171 (5)	0.191 (5)	0.180 (5)	0.090 (4)	0.028 (4)	0.022 (4)
O2	0.230 (6)	0.105 (3)	0.178 (5)	−0.065 (3)	0.087 (4)	−0.028 (3)
O3	0.123 (3)	0.203 (5)	0.252 (6)	0.055 (4)	0.089 (4)	0.031 (4)
N5	0.120 (4)	0.076 (3)	0.105 (3)	−0.009 (3)	0.014 (3)	−0.019 (2)
O1W	0.158 (4)	0.194 (4)	0.132 (3)	−0.037 (4)	−0.025 (3)	0.056 (3)
O2W	0.233 (5)	0.123 (3)	0.113 (3)	−0.002 (3)	0.028 (3)	0.009 (2)
O3W	0.147 (4)	0.169 (4)	0.174 (4)	0.038 (3)	0.039 (3)	0.020 (3)

### Geometric parameters (Å, º)

**Table:**

Ni1—Cl1	2.3035 (9)	C7—C8	1.384 (5)
Ni1—N1	1.989 (2)	C8—C9	1.363 (5)
Ni1—N2	2.088 (2)	C9—C10	1.374 (5)
Ni1—N3	2.107 (2)	C11—C12	1.373 (5)
Ni1—N4	1.983 (2)	C12—C13	1.378 (5)
O1—N5	1.185 (8)	C13—C14	1.372 (5)
O2—N5	1.211 (8)	C14—C15	1.384 (4)
O3—N5	1.227 (7)	C15—C16	1.482 (4)
O1W—H2W	0.8300	C16—C17	1.384 (4)
O1W—H1W	0.8100	C17—C18	1.378 (5)
O2W—H4W	0.8300	C18—C19	1.371 (5)
O2W—H3W	0.8400	C19—C20	1.382 (5)
O3W—H5W	0.8500	C1—H1	0.9300
O3W—H6W	0.8300	C2—H2	0.9300
N1—C5	1.348 (4)	C3—H3	0.9300
N1—C1	1.335 (4)	C4—H4	0.9300
N2—C6	1.350 (3)	C7—H7	0.9300
N2—C10	1.337 (4)	C8—H8	0.9300
N3—C11	1.339 (4)	C9—H9	0.9300
N3—C15	1.348 (3)	C10—H10	0.9300
N4—C20	1.337 (4)	C11—H11	0.9300
N4—C16	1.346 (3)	C12—H12	0.9300
C1—C2	1.370 (5)	C13—H13	0.9300
C2—C3	1.375 (5)	C14—H14	0.9300
C3—C4	1.376 (5)	C17—H17	0.9300
C4—C5	1.387 (4)	C18—H18	0.9300
C5—C6	1.480 (4)	C19—H19	0.9300
C6—C7	1.384 (4)	C20—H20	0.9300
			
Cl1—Ni1—N1	92.75 (7)	C12—C13—C14	119.7 (3)
Cl1—Ni1—N2	128.03 (6)	C13—C14—C15	119.0 (3)
Cl1—Ni1—N3	123.28 (7)	C14—C15—C16	123.1 (3)
Cl1—Ni1—N4	92.10 (8)	N3—C15—C16	115.4 (2)
N1—Ni1—N2	79.96 (9)	N3—C15—C14	121.5 (3)
N1—Ni1—N3	97.75 (9)	N4—C16—C15	114.9 (2)
N1—Ni1—N4	175.13 (10)	N4—C16—C17	120.8 (2)
N2—Ni1—N3	108.69 (9)	C15—C16—C17	124.3 (2)
N2—Ni1—N4	96.75 (10)	C16—C17—C18	119.3 (3)
N3—Ni1—N4	79.84 (9)	C17—C18—C19	119.8 (3)
H1W—O1W—H2W	122.00	C18—C19—C20	118.3 (3)
H3W—O2W—H4W	113.00	N4—C20—C19	122.3 (3)
H5W—O3W—H6W	112.00	N1—C1—H1	119.00
Ni1—N1—C5	116.62 (18)	C2—C1—H1	119.00
Ni1—N1—C1	124.1 (2)	C1—C2—H2	121.00
C1—N1—C5	119.3 (3)	C3—C2—H2	121.00
Ni1—N2—C6	113.39 (18)	C4—C3—H3	120.00
Ni1—N2—C10	128.0 (2)	C2—C3—H3	120.00
C6—N2—C10	118.7 (2)	C3—C4—H4	120.00
C11—N3—C15	118.7 (2)	C5—C4—H4	120.00
Ni1—N3—C11	128.63 (18)	C8—C7—H7	120.00
Ni1—N3—C15	112.66 (17)	C6—C7—H7	121.00
Ni1—N4—C16	117.18 (19)	C7—C8—H8	120.00
Ni1—N4—C20	123.4 (2)	C9—C8—H8	120.00
C16—N4—C20	119.4 (3)	C8—C9—H9	120.00
O2—N5—O3	115.9 (5)	C10—C9—H9	120.00
O1—N5—O2	123.3 (6)	C9—C10—H10	119.00
O1—N5—O3	120.7 (6)	N2—C10—H10	119.00
N1—C1—C2	122.6 (3)	N3—C11—H11	119.00
C1—C2—C3	118.8 (3)	C12—C11—H11	119.00
C2—C3—C4	119.3 (3)	C11—C12—H12	121.00
C3—C4—C5	119.5 (3)	C13—C12—H12	121.00
C4—C5—C6	124.2 (2)	C14—C13—H13	120.00
N1—C5—C6	115.2 (2)	C12—C13—H13	120.00
N1—C5—C4	120.6 (3)	C13—C14—H14	120.00
C5—C6—C7	123.9 (2)	C15—C14—H14	121.00
N2—C6—C5	114.8 (2)	C16—C17—H17	120.00
N2—C6—C7	121.2 (3)	C18—C17—H17	120.00
C6—C7—C8	119.0 (3)	C19—C18—H18	120.00
C7—C8—C9	119.5 (3)	C17—C18—H18	120.00
C8—C9—C10	119.0 (3)	C18—C19—H19	121.00
N2—C10—C9	122.6 (3)	C20—C19—H19	121.00
N3—C11—C12	122.6 (3)	C19—C20—H20	119.00
C11—C12—C13	118.5 (3)	N4—C20—H20	119.00
			
Cl1—Ni1—N1—C1	50.9 (3)	Ni1—N3—C11—C12	−178.4 (2)
Cl1—Ni1—N1—C5	−127.61 (19)	C15—N3—C11—C12	−1.0 (5)
N2—Ni1—N1—C1	179.0 (3)	Ni1—N3—C15—C14	179.4 (2)
N2—Ni1—N1—C5	0.54 (19)	Ni1—N3—C15—C16	−1.8 (3)
N3—Ni1—N1—C1	−73.3 (3)	C11—N3—C15—C14	1.6 (4)
N3—Ni1—N1—C5	108.3 (2)	C11—N3—C15—C16	−179.6 (3)
Cl1—Ni1—N2—C6	84.54 (19)	Ni1—N4—C16—C15	0.4 (3)
Cl1—Ni1—N2—C10	−95.4 (2)	Ni1—N4—C16—C17	179.2 (2)
N1—Ni1—N2—C6	−1.07 (18)	C20—N4—C16—C15	−178.3 (3)
N1—Ni1—N2—C10	179.0 (2)	C20—N4—C16—C17	0.4 (4)
N3—Ni1—N2—C6	−95.92 (18)	Ni1—N4—C20—C19	−178.7 (2)
N3—Ni1—N2—C10	84.2 (2)	C16—N4—C20—C19	0.0 (5)
N4—Ni1—N2—C6	−177.45 (18)	N1—C1—C2—C3	0.7 (6)
N4—Ni1—N2—C10	2.7 (2)	C1—C2—C3—C4	0.1 (5)
Cl1—Ni1—N3—C11	−95.1 (3)	C2—C3—C4—C5	−0.3 (5)
Cl1—Ni1—N3—C15	87.41 (19)	C3—C4—C5—N1	−0.2 (4)
N1—Ni1—N3—C11	3.4 (3)	C3—C4—C5—C6	179.2 (3)
N1—Ni1—N3—C15	−174.12 (19)	N1—C5—C6—N2	−1.0 (3)
N2—Ni1—N3—C11	85.4 (3)	N1—C5—C6—C7	179.1 (3)
N2—Ni1—N3—C15	−92.2 (2)	C4—C5—C6—N2	179.5 (3)
N4—Ni1—N3—C11	179.1 (3)	C4—C5—C6—C7	−0.4 (4)
N4—Ni1—N3—C15	1.59 (19)	N2—C6—C7—C8	−1.1 (4)
Cl1—Ni1—N4—C16	−124.5 (2)	C5—C6—C7—C8	178.8 (3)
Cl1—Ni1—N4—C20	54.2 (2)	C6—C7—C8—C9	−0.2 (5)
N2—Ni1—N4—C16	106.8 (2)	C7—C8—C9—C10	1.3 (5)
N2—Ni1—N4—C20	−74.5 (2)	C8—C9—C10—N2	−1.2 (5)
N3—Ni1—N4—C16	−1.1 (2)	N3—C11—C12—C13	0.0 (5)
N3—Ni1—N4—C20	177.6 (3)	C11—C12—C13—C14	0.4 (5)
Ni1—N1—C1—C2	−179.7 (3)	C12—C13—C14—C15	0.2 (5)
C5—N1—C1—C2	−1.3 (5)	C13—C14—C15—N3	−1.2 (5)
Ni1—N1—C5—C4	179.6 (2)	C13—C14—C15—C16	−179.9 (3)
Ni1—N1—C5—C6	0.0 (3)	N3—C15—C16—N4	1.0 (3)
C1—N1—C5—C4	1.0 (4)	N3—C15—C16—C17	−177.7 (3)
C1—N1—C5—C6	−178.5 (3)	C14—C15—C16—N4	179.8 (3)
Ni1—N2—C6—C5	1.4 (3)	C14—C15—C16—C17	1.1 (4)
Ni1—N2—C6—C7	−178.7 (2)	N4—C16—C17—C18	−0.6 (4)
C10—N2—C6—C5	−178.7 (2)	C15—C16—C17—C18	178.0 (3)
C10—N2—C6—C7	1.2 (4)	C16—C17—C18—C19	0.5 (5)
Ni1—N2—C10—C9	179.9 (2)	C17—C18—C19—C20	−0.1 (5)
C6—N2—C10—C9	0.0 (4)	C18—C19—C20—N4	−0.2 (5)

### Hydrogen-bond geometry (Å, º)

**Table:**

*D*—H···*A*	*D*—H	H···*A*	*D*···*A*	*D*—H···*A*
O1*W*—H1*W*···O3*W*^i^	0.81	2.29	2.876 (6)	129
O1*W*—H2*W*···O2^ii^	0.83	2.18	2.934 (7)	151
O2*W*—H3*W*···O2^ii^	0.84	1.90	2.723 (7)	166
O2*W*—H4*W*···Cl1^i^	0.83	2.47	3.245 (4)	155
O3*W*—H5*W*···O2*W*^iii^	0.85	1.88	2.699 (6)	161
O3*W*—H6*W*···O1^iv^	0.83	2.03	2.839 (7)	165
C14—H14···O2*W*	0.93	2.56	3.424 (5)	155
C18—H18···O1*W*	0.93	2.39	3.257 (6)	156

Symmetry codes: (i) −*x*, −*y*, −*z*; (ii) *x*, *y*, *z*−1; (iii) −*x*+1, −*y*, −*z*; (iv) −*x*+1/2, *y*−1/2, −*z*+1/2.
